# Successful treatment of infectious delayed union after ulnar shortening osteotomy using once-weekly teriparatide with low-intensity pulsed ultrasound

**DOI:** 10.1080/23320885.2021.1894155

**Published:** 2021-03-05

**Authors:** Kiyohito Takamatsu, Takuya Uemura, Ema Onode, Masaru Koshimune

**Affiliations:** a Department of Orthopaedic Surgery, Yodogawa Christian Hospital, Osaka, Japan; b Department of Orthopaedic Surgery, Osaka General Hospital of West Japan Railway Company, Osaka, Japan; c Department of Orthopaedic Surgery, Osaka City University Graduate School of Medicine, Osaka, Japan; d Department of Orthopaedic Surgery, Koshimune Orthopedic Hospital, Osaka, Japan

**Keywords:** Non-union, teriparatide, osteotomy, osteoporosis, infection

## Abstract

We report a 50-year-old woman who presented with infected delayed union after ulnar shortening osteotomy. She was a chronic smoker. Implants were removed and infected tissue was debrided. Sufficient bony union was obtained after 5 months of treatment with weekly teriparatide and low-intensity pulsed ultrasound during the infection-controlled waiting period.

## Introduction

As a treatment for ulnar abutment syndrome, ulnar shortening osteotomy with a plate is widely used, and its postoperative results are satisfactory. However, the possibility of non-union after plate fixation in ulnar shortening osteotomy is small but significant [1]. Chan et al. reported non-union in 4 of 63 patients (6%), and Verhiel et al. detected non-union in 6 of 98 cases (6%) [2,3]. In addition, infectious non-union or delayed union after plate fixation is more difficult to treat. In general, implant removal and sufficient debridement of the infected tissue is necessary. Following adequate postoperative infection control, a second stage of internal fixation with possible bone grafting is often needed that can be extremely challenging [4,5].

This report described a case of infectious delayed union with a loosened screw after ulnar shortening osteotomy that was successfully treated using teriparatide (once-weekly administration) and low-intensity pulsed ultrasound (LIPUS) during the infection-controlled waiting period after implant removal. Bony union was achieved without an additional surgical intervention.

## Case presentation

**Figure 1.**
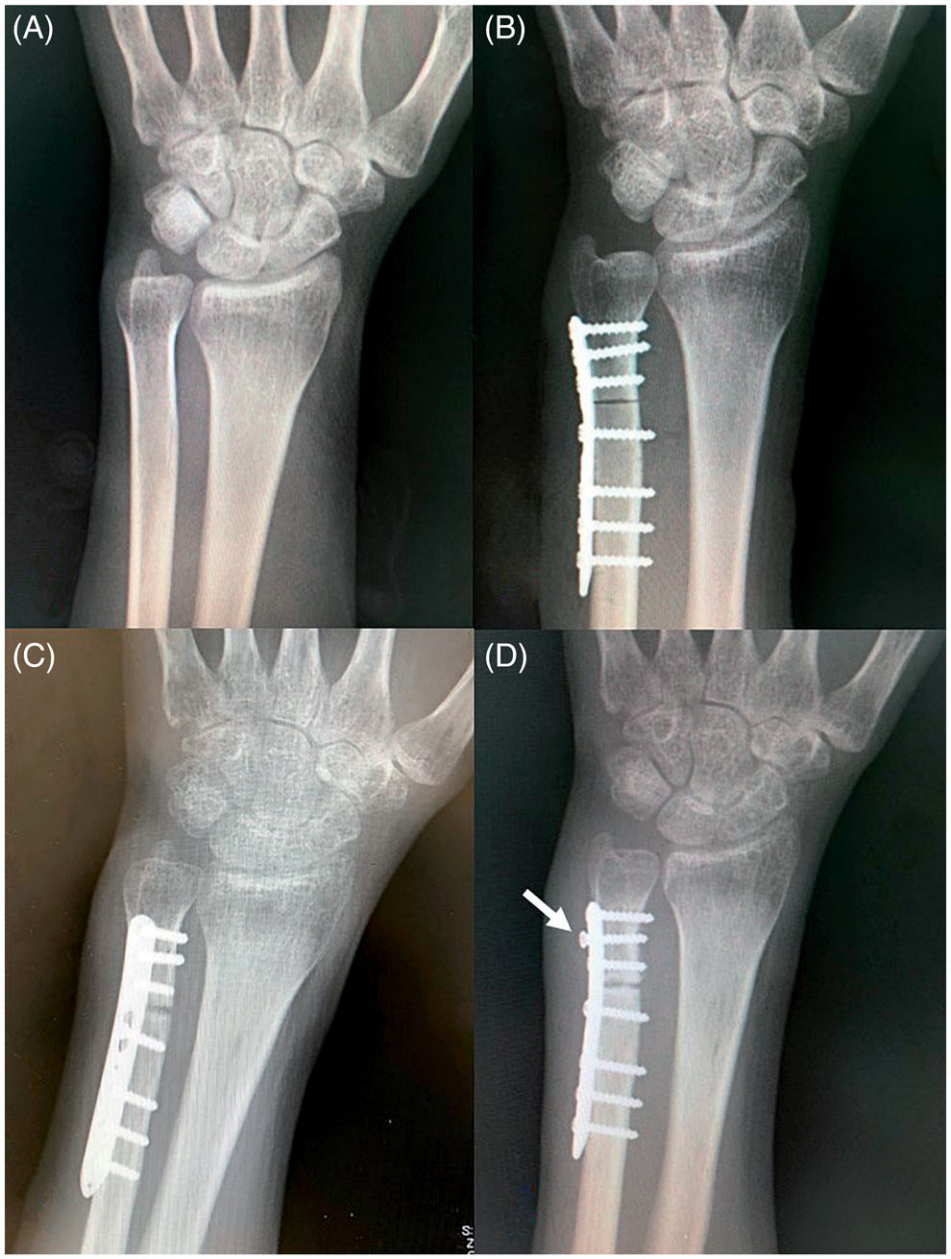
(A) Preoperative X-ray. (B) Postoperative X-ray (immediately after ulnar shortening osteotomy). (C) Postoperative X-ray (3 months after the surgery). (D) Postoperative X-ray (5 months after the surgery). The distal screw was loosened (Arrow). There was a bony absorption at the osteotomy site.

A 50-year-old woman had persistent ulnar wrist pain for over 4 months despite conservative treatment with bracing, physical therapy, and pain medication. The ulnocarpal stress test was positive without distal radioulnar joint instability and tenderness of the extensor carpi ulnaris tendon and pisiform. In addition, there was a positive ulnar variance of 2 mm on the radiograph. She had no trauma or medical history of diabetes mellitus. She had a 30-year history of smoking 20 cigarettes/day. She was diagnosed with ulnar abutment syndrome. Ulnar shortening transverse osteotomy was performed, and a seven-hole locking plate (Meira Co Ltd, Nagoya, Japan) was used for fixation (Figure 1(A,B)). After the surgery, she was placed in a cast for 6 weeks. The initial internal fixation was rigid with zero ulnar variance.

**Figure 2.**
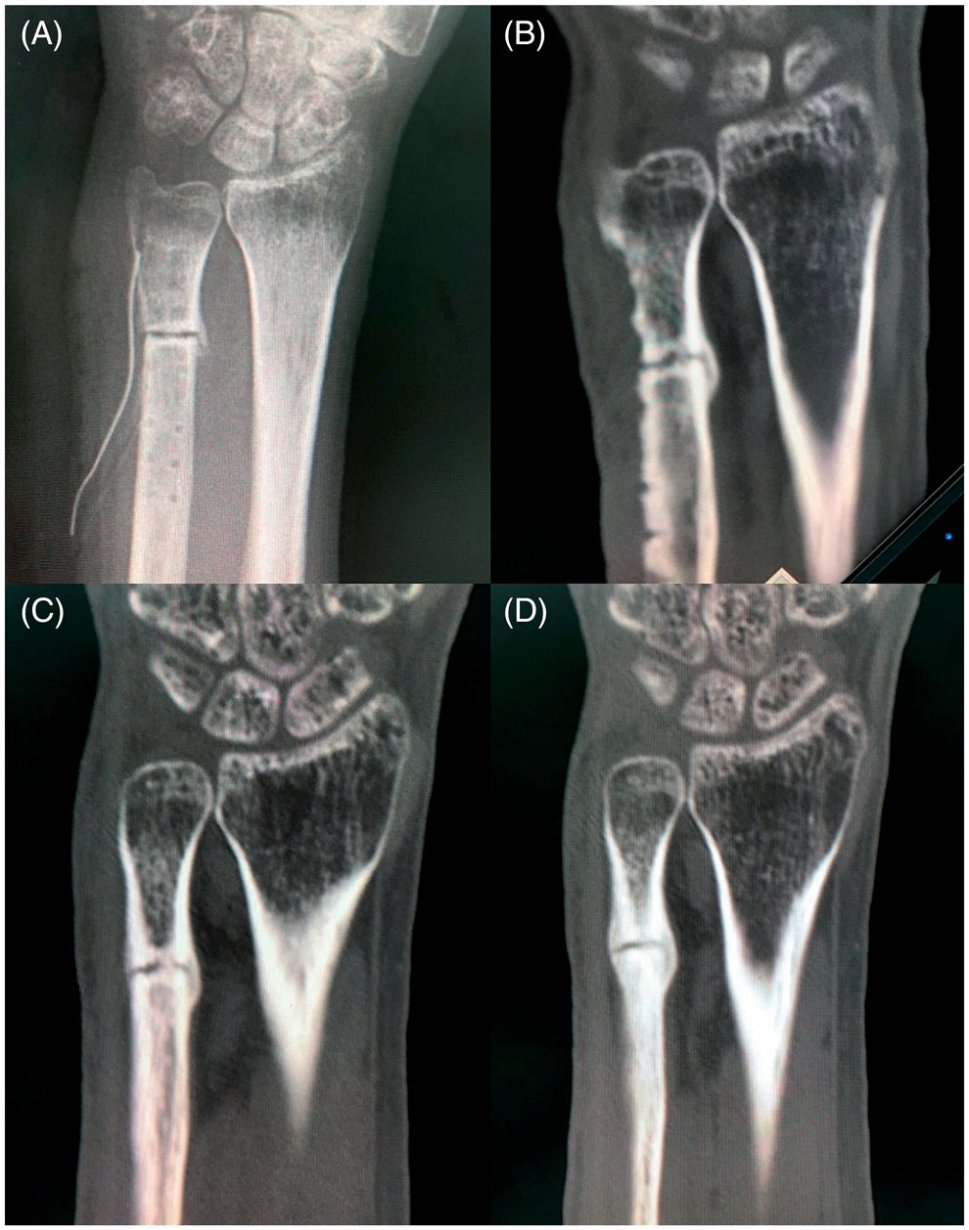
(A) Postoperative X-ray (immediately after implant removal and debridement). (B) Postoperative computed tomography (CT, 1 month after implant removal and debridement). There was poor callus formation and a bony absorption at the osteotomy site. (C) Postoperative CT (4 months after implant removal and debridement and 3 months after the start of teriparatide therapy and low-intensity pulsed ultrasound [LIPUS]). (D) Postoperative CT (5 months after implant removal and debridement and 4 months after the start of teriparatide therapy and LIPUS). Bony union at the osteotomy site was observed.

Five months after the operation, the patient complained of swelling and pain at the surgical site and loosening of the screw and radiolucency at the osteotomy site were observed on X-ray (Figure 1(C,D)). White blood cell count and C-reactive protein (CRP) levels were 9100/µL and 0.02 mg/dL, respectively. In view of the loosened hardware, we removed the plate. However, intraoperatively, abscess formation at the osteotomy site and around the loosened screw was found and no bony union was noted. We diagnosed infected delayed union and proceeded with removal of all screws and plate, followed by thorough debridement of the infected tissue (Figure 2(A)). The pus and infected tissue were cultured, but no growth was observed. After debridement, a short arm splint was applied for 4 weeks and then a wrist brace (Wrist care pro, Alcare Co Ltd, Tokyo Japan) was applied for additional 6 weeks, except during range of motion exercises, because there was some stability even after implant removal. The patient was administered oral linezolid 1200 mg/day for 3 weeks, but an allergic skin rash appeared. Therefore, oral minocycline 100 mg/day was administered for an additional 2 weeks. The allergic reaction to linezolid resolved a week after the drug withdrawal. The laboratory parameters were normalized 16 days after implant removal and debridement (white blood cell count, 7600/μl; CRP, 0.09 mg/dL).

One month after implant removal and debridement, the infection was still under control. However, bony absorption was observed at the osteotomy site on computed tomography (CT) (Figure 2(B)). For the delayed union of the distal ulna, we planned excision of pseudoarthrosis and bone graft after 6 months of normal CRP levels and white blood cell count levels. Low-intensity pulsed ultrasound (LIPUS) was initiated during the waiting period. Additionally, weekly subcutaneous injections of teriparatide (56.5 µg, Teribone; Asahi Kasei Pharma, Tokyo, Japan) were used to accelerate the bone healing and treat the patient’s osteoporosis. After the second surgery of implant removal, the bone mineral density was checked and it was 61% of the young adult mean. In short, LIPUS and teriparatide therapy was started 6 weeks after implant removal and debridement, respectively. LIPUS and teriparatide had been used for 5 months and 12 months, respectively.

Callus formation at the osteotomy site was observed on CT after 3 months after implant removal (Figure 2(C)). Five months after implant removal, the bony union was achieved on CT (Figure 2(D)), and the ulnar wrist pain had disappeared. There were no side effects, such as confusion, constipation, depression, dry mouth, or headache, of teriparatide treatment. Two years after the last surgery, no ulnar wrist pain was observed and the patient’s Mayo Wrist score was 85 with 75%–100% grip strength compared with normal, with a wrist flexion–extension arc of more than 120°.

## Discussion

Using ulnar shortening osteotomy plating systems that exert compression force on the osteotomy site, the rates of non-union can be decreased to 4%–7% [1]. However, Chen et al. reported that the bony union failure rate reached 30% in smokers [6]. Furthermore, the average periods to bony union after ulnar shortening osteotomy were 4.1 and 7.1 months in non-smokers and in smokers, respectively, indicating that the bony union period was significantly prolonged in smokers [6]. The current case report showed that the combination of LIPUS and once-weekly teriparatide resulted in bony union 5 months after implant removal and debridement in a smoker who exhibited infectious delayed union after ulnar shortening osteotomy.

Few studies have described infectious non-union after internal fixation surgery [4,5,7,8]. It is common to remove implants and curette infected tissue and then perform the second stage autologous bone grafting and internal fixation after the infection has subsided [4,5]. However, autologous bone grafting and a second internal fixation at the non-union site may cause the infection to recur [7,8]. To avoid recurrent infection, the secondary surgery should be performed after a sufficient waiting period [5,8]. Therefore, additional surgical treatment is challenging, and it is difficult to achieve bony union in infectious non-union cases. We believe that non-invasive therapy with a combination of weekly teriparatide and LIPUS was beneficial for our patient, even if it took 5 months for complete bony union, although it cost more than secondary surgical treatment and teriparatide treatment had some side effects.

Several clinical studies have reported that LIPUS enhances the healing of fresh fractures, delayed unions, and non-unions [9–11]. A systematic review of healing of fracture non-unions treated with LIPUS showed an overall average success rate of more than 80%, which is comparable to the success of surgical treatment for non-infected non-unions [10]. In contrast, some reports stated that teriparatide was useful for treatment of both fractures and non-union. In 102 postmenopausal women with distal radius fractures, Aspenberg et al. showed that the time to healing was shorter in the 20 μg teriparatide group than in the placebo group [12]. Tamai et al. reported the efficacy of teriparatide in a case of non-union after failed ankle arthrodesis for Charcot arthropathy [13]. In the report, a 12-week course of daily teriparatide (20 μg, subcutaneous injection) resulted in bony union of the ankle arthrodesis. These reports suggest that daily teriparatide can promote callus formation for bony union. Furthermore, once-weekly teriparatide has also been used to treat osteoporotic vertebral fracture, and corrective bony union of the affected vertebra was obtained using this drug at 2 months after injury [14]. When once-weekly teriparatide was used for atypical subtrochanteric femur fractures, union of bilateral femurs was achieved after 3 months [15]. In addition, a report described the efficacy of the combination of once-weekly teriparatide and LIPUS for correcting femoral non-union, with bony union achieved after 6 months [16]. We cannot conclude for sure which is more likely to have caused the improvement in bony union, LIPUS or teriparatide. However, we think teriparatide is more likely to have caused the improvement in bony union than LIPUS. In our previous report, teriparatide was more effective than LIPUS for achieving bony union in the two cases of the non-union in smokers after ulnar shortening osteotomy [17]. Warden et al. reported that teriparatide and LIPUS had contrasting additive, rather than synergistic, effects during fracture healing in rat studies, i.e. teriparatide primarily increased the callus bone mineral content, whereas LIPUS increased the callus size [18]. Therefore, we believe that the combination of LIPUS and teriparatide is the best way to achieve bony union. In the current case, we noted that if the infection can be corrected with appropriate treatment, bony union may be obtained even after infectious delayed union with the once-weekly administration of teriparatide in combination with LIPUS.

## Conclusion

We presented a smoker of infectious delayed union after ulnar shortening osteotomy that was successfully treated using once-weekly teriparatide and LIPUS during the infection-controlled waiting period after implant removal without an additional surgical intervention. This combined therapy might be considered in the patients’ group with infectious delayed union after adequate infection control by debridement and long-term antibiotics and/or the patients’ group with high risk of developing non-union.
